# Machine learning and medicine: book review and commentary

**DOI:** 10.1186/s12938-018-0449-9

**Published:** 2018-02-01

**Authors:** Robert Koprowski, Kenneth R. Foster

**Affiliations:** 1 Department of Biomedical Computer Systems, Faculty of Computer Science and Materials Science, Institute of Computer Science, University of Silesia, ul. Będzińska 39, Sosnowiec, 41-200 Poland; 2 Department of Bioengineering, University of Pennsylvania, Philadelphia, USA

**Keywords:** Machine learning, Algorithms, Biomedical technologies, Spreadsheets, Overfitting, Leakage, Practice guidelines

## Abstract

This article is a review of the book “Master machine learning algorithms, discover how they work and implement them from scratch” (ISBN: not available, 37 USD, 163 pages) edited by Jason Brownlee published by the Author, edition, v1.10 http://MachineLearningMastery.com. An accompanying commentary discusses some of the issues that are involved with use of machine learning and data mining techniques to develop predictive models for diagnosis or prognosis of disease, and to call attention to additional requirements for developing diagnostic and prognostic algorithms that are generally useful in medicine. Appendix provides examples that illustrate potential problems with machine learning that are not addressed in the reviewed book.

**Electronic supplementary material:**

The online version of this article (10.1186/s12938-018-0449-9) contains supplementary material, which is available to authorized users.

## Book details

Title: “Master Machine Learning Algorithms, Discover How They Work and Implement Them From Scratch”

Edited by: Jason Brownlee

Published by: Jason Brownlee in 2017. Available online in several editions with varying amounts of supplementary material, cost between $USD 37 and 237. The reviewed edition costs $USD 37, 163 pages.

Machine learning is the subject of a large and sophisticated professional literature, with excellent books for biomedical engineers [1, 2] as well as at least one excellent text available free online [3]. Machine learning, together with related topics such as data mining, provides a set of tools with a huge potential range of applications from improving medical diagnosis to optimization of cancer therapy. It has also been the subject of considerable hype in the popular literature.

The first part of this commentary reviews an introduction to machine learning, “Master Machine Learning Algorithms” which is subtitled “Discover How They Work and Implement Them From Scratch”. The author, Jason Brownlee, aims to introduce readers to practical use of machine learning. On his website (http://machinelearningmastery.com/about/) Brownlee describes himself as a software developer who initially taught himself machine learning “to figure this stuff out”. He also is an active blogger on machine learning, and has written several books on the topic for novices, some available online at his website and others available through online stores such as Amazon. In a sense, Brownlee is one of us, with a Ph.D. (Swinburne University, Melbourne, Australia) and a thesis and academic publications on modeling of artificial immune systems.

*Master Machine Learning Algorithms* can be purchased online at https://machinelearningmastery.com/master-machine-learning-algorithms/ (accessed on 03.08.2017) at modest cost ($USD 37), which also includes 17 Excel spreadsheets to illustrate the main algorithms. His website offers 10 related books (including four at a more advanced level) that are tailored for use with the statistical program such as R or the data mining program Weka (both freely distributed on the internet). So, for very little money, a reader can have a useful basic introduction to the topic together with ready-made software to play around with. Brownlee frequently sends emails to a wide distribution list with interesting tutorial material about topics in machine learning.

In the 162 pages of the version presently being reviewed, Brownlee describes 11 basic machine learning algorithms and implements them in Excel spreadsheets, in a rudimentary but informative way. For each algorithm, the author describes the underlying mathematics, and for most of them he provides a tutorial with links to an Excel spreadsheet and graphs and tables with results. The book is divided roughly into three parts: linear algorithms (pages from 25 to 71), nonlinear algorithms (pages from 72 to 125), and ensemble algorithms (pages from 126 to 149). The algorithms discussed include linear regression, logistic regression, discriminant analysis, classification and regression trees, Naive Bayes, k-nearest neighbours, support vector machines, decision trees. Introductory and concluding chapters discuss general aspects of machine learning, including problems of overfitting.

Obviously, this book is not competitive with other well-known introductions to machine learning for professionals [1–3], nor is it intended to be. In spirit, it is a bit like the introductory book on French cooking entitled *Je Ne Sais Pas Cuisiner* (“I don’t know how to cook”) (Flammarion, 1997)—a collection of recipes and rudimentary instructions for novice cooks but hardly competition for Escoffier or even Julia Child. However, it is very clearly written and for what it tries to accomplish it succeeds well.

We continue with a more detailed review of the book, and conclude with a commentary on some of the larger issues that are involved in applying machine learning and data mining to biomedical problems.

### Where it succeeds

The book nicely fills the gap between popular oriented, often hyperbolic introductions to machine learning for laypeople, and textbooks for professionals. To a novice entering the field, it is highly educational to use the tools of machine learning as provided in Microsoft Excel spreadsheets and trace their operation step by step. Unlike other introductions to machine learning [3–6], the reader does not need to buy expensive software such as Matlab or grapple with complicated software such as R and Weka which are referenced in other versions of this book. This approach has great merit as an introduction to a challenging subject that requires a low initial investment. It is a bit like introducing elementary school students to music by teaching them to play inexpensive recorders: the lessons can instil a lifelong appreciation of music but nobody pretends to turn the kids into musicians. This book will not “make developers awesome at machine learning” as Brownlee’s slogan in his email signature says. Rather, it is a “gentle introduction” (his expression) to a complex field, and is very suitable for helping high school and undergraduate university students get off to a good start with these methods.

### Where it is lacking

For professional use, the major limitation is lack of depth. The 227 word section entitled “how to limit overfitting” mentions standard techniques such as k-fold cross validation, but does not explain how to do it properly. Each algorithm is described in 3–4 pages that are clearly written but lack mathematical detail.

Moreover, the educational value of the book is stymied by a complex programming style in the Excel spreadsheets that will be very hard for novices to follow and adapt to their own problems. For example, cell (173, J) in the spreadsheet 14-SupportVectorMachine.xlsx contains the statement: = IF($H173 < 1;((1 − $E173)*G173 + (1/($A$17*$A173))* $D173* C173);((1 − $E173)*G173))”. The book would be more useful if it the spreadsheets were more easily adapted to other problems. A simpler, if less compact, programming style would enhance the tutorial values of the spreadsheets, as would a closer tie of the spreadsheets to the mathematics in the background discussion.

### The larger problem

Machine learning and data mining techniques can discover previously unknown regularities in data and make useful predictions. But finding regularities in an existing set of data and making useful predictions about data collected in the future are two different things. If we could learn patterns in stock market data and use them to successfully predict the future prices of stock we would all be rich. Building models for use in medicine raises further complications in meeting the needs of physicians and their patients. The following discussion pertains equally to machine learning and data mining, which are closely related.

At the technical level, machine learning and data mining algorithms are now included in numerous software packages and are very easy to use. However, they can be unreliable in the hands of naïve practitioners—just the people to whom this volume is addressed. The problem is that they provide great flexibility in analysis at the cost of interpretability, and thus appear as “black boxes” to an unsophisticated user.

Two problems in particular can easily trip up a novice: overfitting and leakage. Overfitting refers to the tendency of overly complex models to “learn” noise resulting in loss of generalizability (a simple example is fitting a set of data to a high-level polynomial). Leakage occurs when the user inadvertently carries information from the training set (used to train the classifier) to the test set (used to validate the classifier).

Brownlee gives helpful advice about overfitting in several places but does not make it clear how subtle the problem can be. Brownlee does not discuss leakage in this book, although he provides insightful discussions of the problem in his blog (http://machinelearningmastery.com/data-leakage-machine-learning/); an extensive professional literature exists on the subject (e.g. Kaurman 2012). A common novice error is to tune a classifier to obtain the “best” results, but continue to use the same test data—which consequently invalidates its statistical independence and makes it unreliable for validation purposes. There are, of course, many discussions of these problems in the professional literature but these are more advanced sources than this present volume.

A different set of problems arise with developing sophisticated statistical methods for use in clinical medicine. These need to work at the technical level that is familiar to engineers, and also meet the needs of doctors and patients. A quick search on Google Scholar will uncover hundreds of papers that use machine learning or data mining to develop methods to diagnose disease, estimate a patient’s prognosis from a disease, or another purpose. The projects range from, at the high end, a handful of large studies supported by companies such as Google and Apple, to a great many much smaller studies by engineers from around the world. A large fraction of these papers are published in engineering and computer science journals as opposed to practice-oriented medical journals, and are clearly aimed at other engineers.

### Developing useful clinical tests using machine learning

A useful perspective is provided in the widely-cited 1991 paper by Fryback and Thornbury on the efficacy of diagnostic imaging. While the article focuses on diagnostic imaging, similar considerations apply to a wide range of other medical applications.

Fryback and Thornbury emphasize that the medical value of a diagnostic test needs to be assessed on several levels: (1) the technical level; (2) its diagnostic accuracy measured in terms of sensitivity and specificity; (3) its contribution to changing the diagnostic thinking of a physician; (4) its contribution to developing a patient’s management plan; (5) its contribution to improving the patient’s outcome; and (6) the societal costs and benefits of the test.

We consider two examples: machine learning/data mining to diagnose coronary artery disease, and for estimating prognosis of survival from breast cancer. Numerous papers are easily located on Google Scholar on these topics, a large fraction of which appeared in engineering or computer science journals. We describe databases that have been used for such purposes. Our goal is not to criticize the studies, but to point to the differences in scale of data needed to develop an algorithm and in establishing its clinical efficacy for real-world medical use.

Two datasets, available online, have been used to develop algorithms for diagnosis of coronary artery disease (CAD). One is the “Z-Alizadeh” dataset [7] that consists of 55 different clinical parameters, demographic data and results of medical tests measured in 303 patients that were collected from random visitors to a Tehran cardiology center. A second dataset is “heart” (http://www-bcf.usc.edu/~gareth/ISL/data.html), that has 13 attributes from 303 patients from an unknown medical center. This latter data set has been used in an extensive case study in the James’s textbook [3].

Both datasets raise interesting technical issues. They are both unbalanced (unequal numbers of healthy and diseased subjects) and contain a mix of qualitative and quantitative data. Both datasets have too many attributes relative to the number of subjects and must be pruned (choosing a subset of attributes for the classifier). James et al. [3] and Alizadehsani [7] both give excellent discussions of the pruning process, one from the perspective of a research paper and the second from a didactic perspective. One of the attributes in the “heart” data set is the result of the thallium stress test, which is a diagnostic test for CAD. Not surprisingly, James et al. [3] show that this attribute has by far the greatest importance in training a classifier for diagnosis of CAD.

The second example is prognosis of breast cancer survival. Several papers use the Haberman Survival dataset (http://archive.ics.uci.edu/ml/datasets/Haberman’s+Survival), that contains the 5-year survival status of 306 patients who had undergone breast cancer surgery. The data set has two classes (alive or dead 5 years after surgery) and three attributes (age of patient at time of operation, year of patient’s operation, and the number of positive axilliary nodes detected). This data set is also interesting as a didactic example of machine learning for binary classification, and has been discussed by one expert as a particularly difficult problem in binary classification [8] (For comments on that see Appendix and Additional file 1). However, it lacks information such as grade of the tumor and data about hormone sensitivity and use of any adjuvant therapy (such as chemotherapy after surgery) that would be needed for accurate prognosis. The data set is also unbalanced (most of the patients were still alive after 5 years) and it has too few attributes to benefit from the distinctive benefits of machine learning, which is to discover new parameters or combinations of parameters that would improve diagnosis. (Shelby J. Haberman, who collected the data for a 1976 paper on log-linear models, became a distinguished statistician and spent much of his later career at the Educational Testing Service in Princeton NJ).

All three datasets are readily available online, and can be easily imported into statistical programs such as R for use with their built-in machine learning or data mining tools. These three datasets, among others, have been used in a rich research literature, almost entirely focused on algorithm development. But the authors have not always distinguished clearly between technical goals (developing algorithms for classifiers) and actual medical use, using terms such as “survival prediction” or “diagnosis” without qualification. This distinction is understandably, not discussed in Brownlee’s book, or in most other texts on machine learning for that matter.

The differences in scale between an engineering study on algorithm development and a developing a classifier or other mathematical model that is suitable for use in medical practice can be very large.

For example, in cardiology, physicians would need more than a binary classification of a patient as having or not having CAD. Their needs include assessing patients who present with symptoms of stable ischemic heart disease, assessing the extent of the disease, if any, estimating the risk of sudden cardiac death, or choosing optimal treatment strategies. Without strong clinical evidence, few doctors would use a classifier based on clinical indications in lieu of conventional diagnostic methods for detection of CAD, for example coronary CT angiography.

A more plausible use of a classifier would be to calculate pre-test probability to de-select patients from expensive tests that they are unlikely to benefit from. That also would require well controlled clinical studies to justify its use, and it seems that few such studies have been done with classifier based tests. A 2017 systematic review [9] concluded that mathematical models for pre-test prediction of outcomes of tests for stable CAD in cardiology had “only modest success”. No machine learning-based models met the inclusion criteria for acceptance in that review A 2017 study by Korley et al. [10] assessed use of clinical risk factors (such as in the Z-Aldesani database) for diagnosing CAD as a pre-test selection tool. That study derived a classifier used a regularized regression method, based on a derivation set of 679 patents to train and validate a classifier, with additional validation on 1056 patients from a different cohort. The investigators concluded that “clinical risk factors, either individually or in combination, are insufficient for accurately identifying suspected ACS (acute coronary symptom) patients harboring undiagnosed significant coronary artery disease.” The possibility exists, however, that an improved classifier after proper validation might prove more successful.

Estimating prognosis for survival from breast cancer is important in treatment planning and for patient information. A recent model, based on a Cox proportional hazard model, is currently used for estimating prognosis of breast cancer patients after surgery (Wishart et al. 2010 [11]). The model was developed from a cohort of 5694 women who had surgery for invasive breast cancer, and validated using an independent data set of 5468 patients from another medical center. One particular use of this model is to assess probable benefits to a patient from adjuvant therapy.

Overall, the contribution of machine learning or data mining to medical diagnosis to date has been mixed. In their recent systematic review of the development of risk prediction models from electronic health records data, Goldstein et al. [12] noted the potential usefulness of such studies, but also considered areas in which improvement is needed. These include the need for studies to validate their results across different healthcare centers, develop better methods to deal with missing data, and assessing how the algorithms impact clinical decision making.

In a recent tutorial [13] Goldstein et al. describe the use of machine learning to predict risk of death in patients admitted to an emergency after sudden myocardial infarction, using electronic health records of 1944 patients—a data set that is nearly seven times larger than the Z-Alizadehsani dataset [7] but not out of range of many biomedical engineering groups. The authors conclude that machine learning methods “can be employed to help confront issues of multiple and correlated predictors, non-linear relationships, and interactions between predictors and endpoints, in large datasets. However, when using machine-learning methods, extra care is needed in the form of model validation.” The authors recommended a series of practical steps to improve the reliability of machine learning models, and stress the need to test the full range of the modeling process including variable selection. Similar cautionary advice was given by Cruz and Wishart in their 2006 review of the applications of machine learning to estimating cancer prognosis [14]. They noted that “it is clear that machine learning methods can be used to substantially (15–25%) improve the accuracy of predicting cancer susceptibility, recurrence and mortality” but they also complained that “a number of published studies also appear to lack an appropriate level of validation or testing.”

All this calls for more extensive validation of classifiers than engineers would typically contemplate when developing machine learning algorithms. Moreover, evaluation studies should be done in concordance with professional recommendations for conducting and reporting machine learning studies for predictive use in medicine (e.g. Luo et al. 2016 [15]). This requires a higher level of sophistication than can be gained from Brownlee’s otherwise excellent book. For soon-to-be biomedical engineers just entering the field, this book is a useful beginning but they will need to know much more about how to make technology work in medicine [16].

### Additional file

**Additional file 1.** Examples that illustrate potential problems with machine learning.

## Figures and Tables

**Fig. 1:**
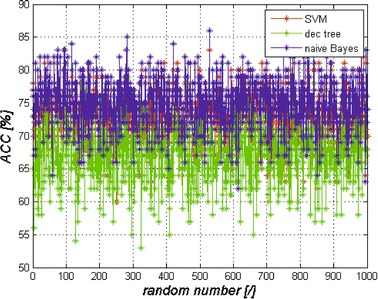
Graph of dependence of accuracy (*ACC*) for 1000 drawings of data for learning and test vector for different classifiers

**Fig. 2:**
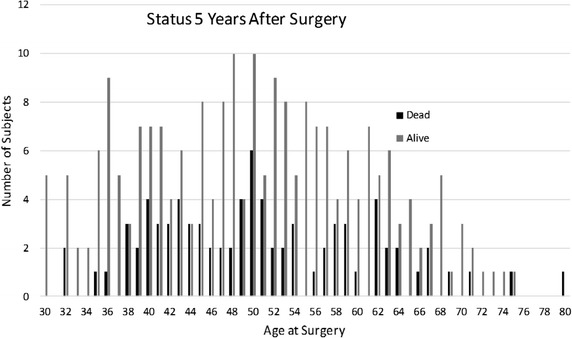
Distribution of ages at time of operation vs. status after 5 years

## Appendix

To illustrate potential problems in applying machine learning methods to biomedical data sets, a series of calculations were done using the Haberman data set [17] (supplementary material). The data set contains 306 cases, women who had undergone surgery for breast cancer between 1958 and 1970 at the University of Chicago’s Billings Hospital [18]. It has three features (year of operation, age of patient at time of diagnosis, and number of nodes) and two classes (patient alive or dead 5 years after surgery)

The Haberman data set [17] has no present day medical value for estimating prognosis, since it lacks important information such as tumor grade, hormone sensitivity, or description of adjuvant therapy. Moreover, it has far too few features to permit effective use of some methods in machine learning such as pruning of decision trees. However, it provides a simple test case for discussions of machine learning (e.g. https://www.kaggle.com/gilsousa/haberman-s-survival).

For this Appendix, the data set was randomly divided into a learning set comprising 2/3 of the complete data (204 cases) and a test set with the remaining 102 cases. Classifiers were trained and validated using three algorithms in the Matlab Statistical Toolbox (support vector machine (SVM), pruned decision trees and naive Bayes classifier). For each algorithm, this process was repeated 1000 times using randomly chosen training and test sets from the dataset. There was no attempt to address problems arising from the significant imbalance in the data set (66% of the patients were still alive 5 years after surgery).

Table 1 summarizes the results for 3 different classifiers, applied repeatedly to the dataset 1000 times. The mean accuracy of all three classifiers ranged from 67.6% for the Naïve Bayes classifier to 73–74.7% for the other two classifiers (Fig. 1). This performance was not impressive, in part because of the very small number of attributes, but nevertheless was considerably better than one could get by assigning outcomes by chance (50% accuracy).

**Table 1:** Summary of the mean, minimum and maximum values of accuracy for 1000 drawings of data for the learning and test vector for various classifiers

Type of classifier	Min (*ACC*)	Mean (*ACC*)	Max (*ACC*)
Support vector machine (SVM)	60	73.0	84
Pruned decision tree	53	67.6	79
Naive Bayes classifier	62	74.7	86

A closer look at the data set shows that, while the two classes have very similar characteristics (Table 2), the age distributions have subtle differences, particularly at their ends (Fig. 2). A calculation of 5-year survival probability from the dataset (Table 3) shows that the 5-year survival of the older women was lower than for younger patients, although the differences were small and perhaps not statistically significant (Table 3). By contrast, the number of tumor nodes among the 5-year survivors at time of surgery was slightly higher than among the decedents (2.8 vs. 2.0), which is in the unexpected direction. However, the distribution of this attribute was about the same for both groups, and this attribute contributed little to the training of the classifiers.

**Table 2:** Characteristics of subjects in Haberman data set

	Class 1 (patient alive 5 years after operation) (n = 204) (mean ± S.D.)	Class 2 (patient died within 5 years of operation) (n = 102) (mean ± S.D.)	*p*
Mean date of operation	Aug. 1962 ± 3.2 years	Aug. 1962 ± 3.3 years	
Patient age at time of operation (mean ± S.D.)	52.0 ± 11.0	53.7 ± 10.2	0.19
Number of nodes (mean ± S.D.)	2.8 ± 5.9	2.0 ± 9.2	0.36

**Table 3:** Probabilty of 5-year survival as function of age at time of surgery (from Haberman data set)

Age range	Subjects	Alive after 5 years	Probability alive at 5 years
30–34	14	12	0.86
35–39	26	24	0.92
40–44	40	28	0.70
45–49	44	29	0.66
50–54	56	38	0.68
55–59	43	35	0.81
60–64	35	26	0.74
65–69	27	18	0.67
70–74	16	12	0.75
75–79	4	3	0.75

We conclude that the classifiers are training chiefly on small differences in the age distribution in survivors vs. decedents, particularly at the ends of the distributions. This is likely to make the results very fragile, i.e. difficult to replicate with different data sets.

The medical significance of these classifiers with that data set are unclear. A higher 5-year mortality among the older women may result from a greater vulnerability to the disease or to complications of treatment. Or it may reflect a higher noncancer mortality rate in the older women. Apart from choosing younger patients, it is difficult to envision how physicans could use these results to improve treatment of their patients.

The above discussion shows the need to consider both the mechanics of developing classifiers, as well as the medical context and requirements for their use. Students reading Brownlee’s fine book will get a feeling for the first of these, but they need a sophisticated understanding of the second as well.

## Abbreviations

S.D.: standard deviation
CAD: coronary artery disease
SVM: support vector machine
ACS: acute coronary symptom
