# Supplementary material for: Machine learning and medicine: book review and commentary
Source: Biomed Eng Online. 2018 Feb 1;17:17. doi: 10.1186/s12938-018-0449-9 (PMC5805011; doi:10.1186/s12938-018-0449-9)
Supplement: Supplementary file 1 — Additional file 1. Examples that illustrate potential problems with machine learning. [file 12938_2018_449_MOESM1_ESM.pdf]

Below we give 6 further examples implemented for 3 different classifiers that are related to biomedical engineering. The discussion below is rather technical and might be skipped by readers who only want an overview of Brownlee's fine book.

- support vector machine (SVM), decision trees and naive Bayes classifier. Binary decision trees were induced with the CART algorithm with Gini index. Full decision trees and then (depending on the needs) pruned decision trees were induced. The pruning criterion was the smallest cross-validation error. In the subsequent examples, the following problems were analysed:

1. Limiting the number of features in relation to the size of the learning and test vector,
2. Leaking the test data into the training data,
3. Leaking the correct prediction or ground truth into the test data,
4. Inclusion of data not present in the model's operational environment,
5. Distorting information from samples outside of scope of the model's intended use,
6. Deliberate limiting the length of the test vector,

In every case the accuracy value (*ACC*) was assessed defined as $ACC=(TP+TN)/(TP+TN+FN+FP)$ where *TN* - true negative, *TP* - true positive, *FN* - false negative, *FP* - false positive. For the sake of transparency, the obtained results were not further statistically analysed in the article.

The obtained results of classification were realized for two independent groups of data.

First group was constituted by artificial and random values of features and variable lengths of learning and test vectors (if they occurred). The random number generator allowed the drawing of values in a uniform interval in the range from 0 to 1.

The second group of input data for classification was constituted by the data from Heberman's Survival Data Set from UCI Machine Learning Repository databases [**2**]. The dataset contains cases from a study that was conducted between 1958 and 1970 at the University of Chicago's Billings Hospital on the survival of patients who had undergone surgery for breast cancer [**3**], [**4**]. The data contained four features for 306 cases. The respective symbols of attributes and their description:

- $w(1)$ - age of the patient at the time of operation (numerical),
- $w(2)$ - patient's year of operation (year - 1900, numerical),

- *w*(3) - number of positive axillary nodes detected (numerical).

As a result there were two classes as ground truth:

- the patient survived 5 years or longer (first class) or
- the patient died within 5 year (second class).

In all cases in question the order of cases in the learning and test vectors was random. The developed test software was implemented in a computer with an Intel® Core i7 processor - 3770 CPU 3.4 GHz, 10 GB of RAM with the operational environment Matlab Version 7.11.0.584 (R2010b) Java VM Version: Java 1.6.0_17-b04 with Sun Microsystems Inc. Java HotSpot(TM) 64-Bit Server VM mixed mode. Additionally, Statistics Toolbox Version 7.4 (R2010b) was used.

First example

The data were generated randomly. The length of the learning vector *u* was changed in the range from 4 to 50. The number of features *k* was changed in the range from 4 to 50. The initial value (4) is the bottom line for correct functioning of classification in Matlab. The upper line (50) was adopted arbitrarily. The values of features were drawn according to the uniform distribution in the range from 0 to 1. The obtained results, the change in the accuracy value (*ACC*), are shown in Fig. 1 and Fig. 2 for the SVM classifier, Fig. 3 and Fig. 4 for the classifier being the binary decision tree and in Fig. 5 and Fig. 6 for the naive Bayes classifier.

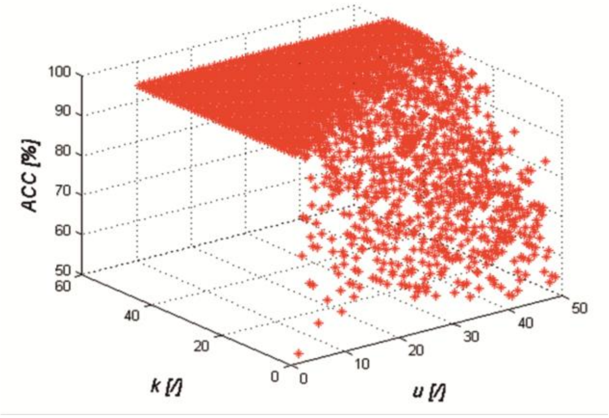

Fig.1 Graph of dependence of accuracy (*ACC*) for various number of features and for different lengths of the learning vector - SVM classifier

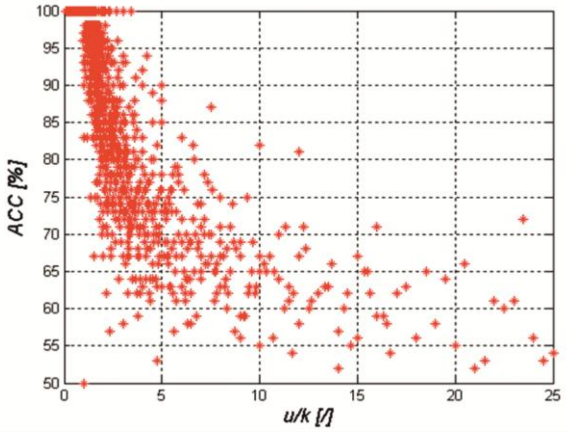

Fig. 2. Graph of dependence of accuracy (*ACC*) for various values of the ratio *u/k* - SVM classifier

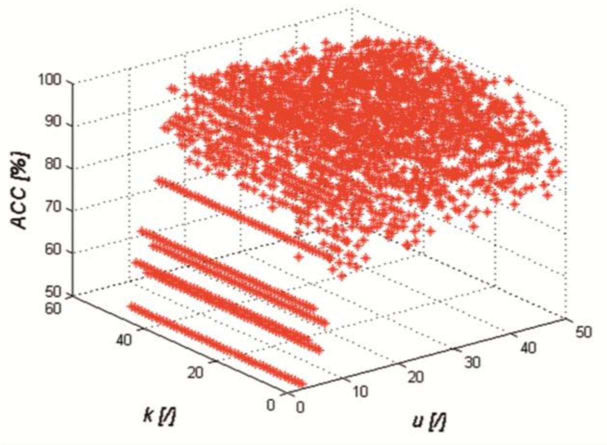

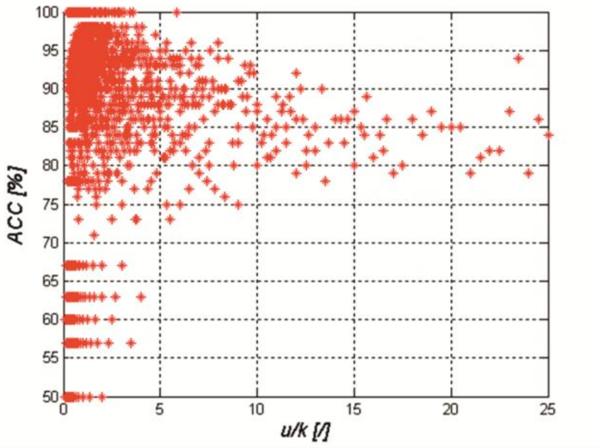

Fig.3. Graph of dependence of accuracy (*ACC*) for various numbers of features and for different lengths of the learning vector - full decision tree.

Fig. 4. Graph of dependence of accuracy (*ACC*) for various values of the ratio *u/k* - full decision trees

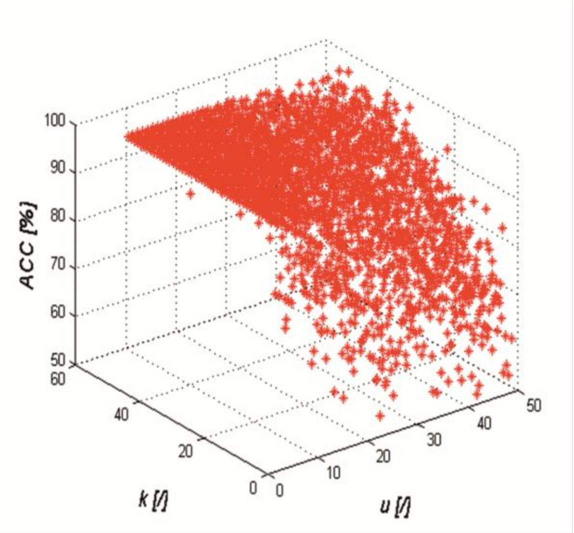

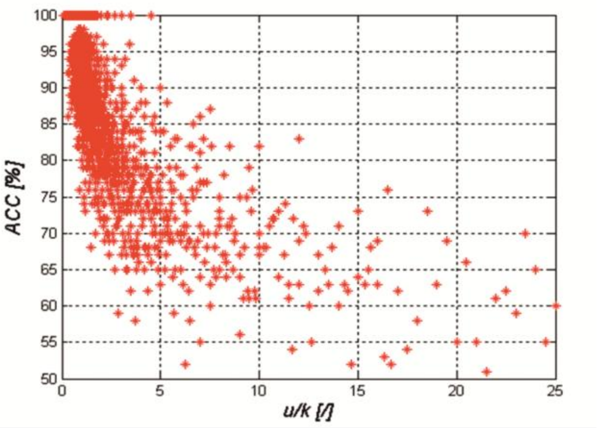

Fig.5. Graph of dependence of accuracy (*ACC*) for various numbers of features and for different lengths of the learning vector - naive Bayes classifier

Fig.6. Graph of dependence of accuracy (*ACC*) for various values of the ratio *u/k* - naive Bayes classifier

The obtained results are specific and dependent on the type of classifier as well as on the ratio of the number of features to the length of the learning vector. Similar results were obtained for the SVM and naive Bayes classifier. The results for decision trees are distinct due to the lack of pruning and visible overfitting. It results not only in exaggerated values of accuracy but also in the lack of practical usefulness of the results. The other types of classifiers (SVM and naive Bayes classifier) have the accuracy value at the level of ≈50%, obtained for the ratio *u/k* equalling 5 or more. It indicates that the length of the vector must be

at least 5 times higher than the number of features, which is according to the commentary presented in [**1**].

Second example - the choice of the 'appropriate' learning and test vector

In this examples as well as in the subsequent ones (unless otherwise indicated), the real data (Haberman's Survival Data Set) were used divided into the learning vector (2/3 of complete data - 204 cases) and the test vector (1/3 - 102 cases). A drawing was conducted 1000 times to establish which data should be assigned to the learning vector and which to the test vector (the length of the learning and test vector is constant). The results for 3 different types of classifiers (SVM, pruned decision trees and naive Bayes classifier) are shown in Fig. 7.

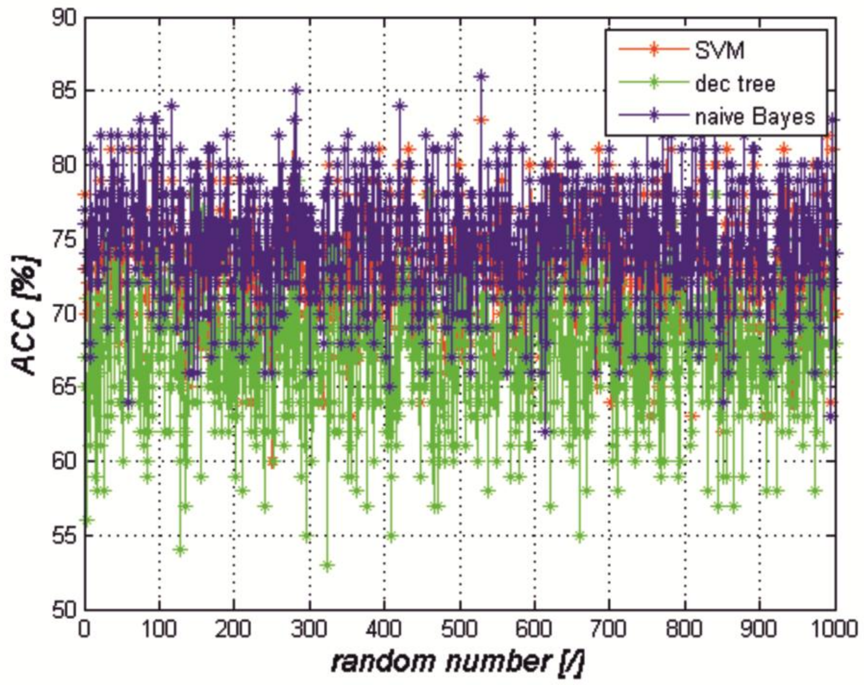

Fig. 7 Graph of dependence of accuracy (*ACC*) for 1000 drawings of data for the learning and test vector for different classifiers.

Tab.1 Summary of the mean, minimum and maximum values of accuracy for 1000 drawings of data for the learning and test vector for various classifiers.

**Type of classifier**	min (*ACC*)	mean (*ACC*)	max (*ACC*)
SVM	60	73.01	84

Pruned decision tree	53	67.6	79
Naive Bayes classifier	62	74.7	86

As it can be seen form the graph (Fig. 7.) appropriate drawing may be found in order to obtain the best results. Therefore, fully correctly and in conformity with all rules, the obtained results may be influenced - according to Tab. 1.

As it is shown in Tab.1, the values of accuracy change by about 33% depending on the chosen classifier and type of drawing (See Fig. 7).

Third example - Leaking test data into the training data

Data leakage still will be simulated by multiplication of data between the learning and test vector. The multiplication of data (Fig. 8) will concern the percentage contribution of training data *q* from the value 0% to 100%. The length of both the learning and test vector does not change (learning vector - 204 cases and test vector - 102 cases). The results for changes in the *q* value by every 0.1% are shown in Fig.9.

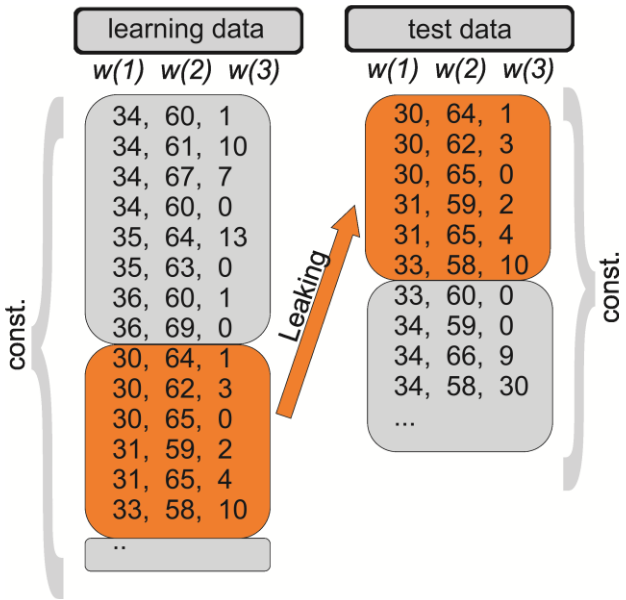

Fig. 8. Block diagram of data multiplication between the learning and test vector

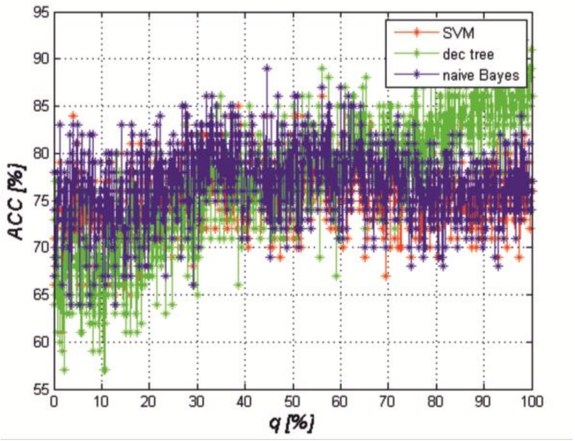

Fig. 9. Graph of changes in accuracy for subsequent values of *q* coefficient for 3 tested classifiers

Fourth example - Leaking the correct prediction or ground truth into the test data

The leakage of the ground truth data to prediction results allows for any kind of influence on the obtained results. In this case, maintaining the length of the test and learning vector in the proportions 1/3 to 2/3 simultaneously changed the percentage ground truth data leakage to prediction results - determined as a *v* coefficient (Fig. 10). The range of the coefficient *v* value was being changed in the range from 0 to 100% every 1%. The obtained results are shown in Fig. 11.

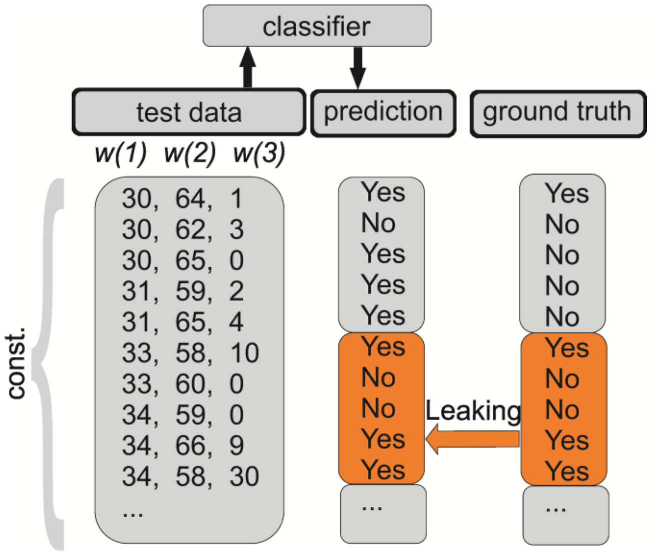

Fig. 10. Block diagram of data multiplication between the learning and test vector.

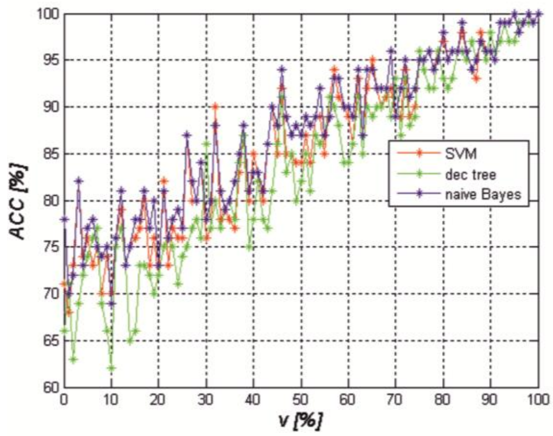

Fig. 11. Graph of accuracy changes for subsequent values of *v* coefficient for three tested classifiers.

As it was expected, the bigger ground truth data leakage to the prediction results, the seemingly bigger efficacy of the classifier (accuracy value).

Fifth example - Inclusion of data not present in the model's operational environment

The test of classifier in the correct implementation should be conducted for the test data whose range of variability of particular features is the same or similar to the learning data. In this example, the data vector was divided into the learning data and test date in different proportions. These proportions were dependent on the mean value of particular features. The learning vector consisted of cases for which the values of the first feature (*w*(1)) were higher than its mean value. The test vector instead comprised the remaining value for which the

values of the first feature were lower than its mean value. By analogy, the other two feature *w*(2) and *w*(3) were tested - Fig. 12. The obtained results are shown in Tab.2.

First of all, it should be concluded that in none of the examples (second, third and fourth) it was possible to obtain such bad results i.e. minimum value of accuracy 49% for SVM and 51% for the pruned decision tree and naive Bayes classifier. Secondly, in most of the other examples, the results are much worse (by about a few percent) than in the case of a classical division into the learning and test vector.

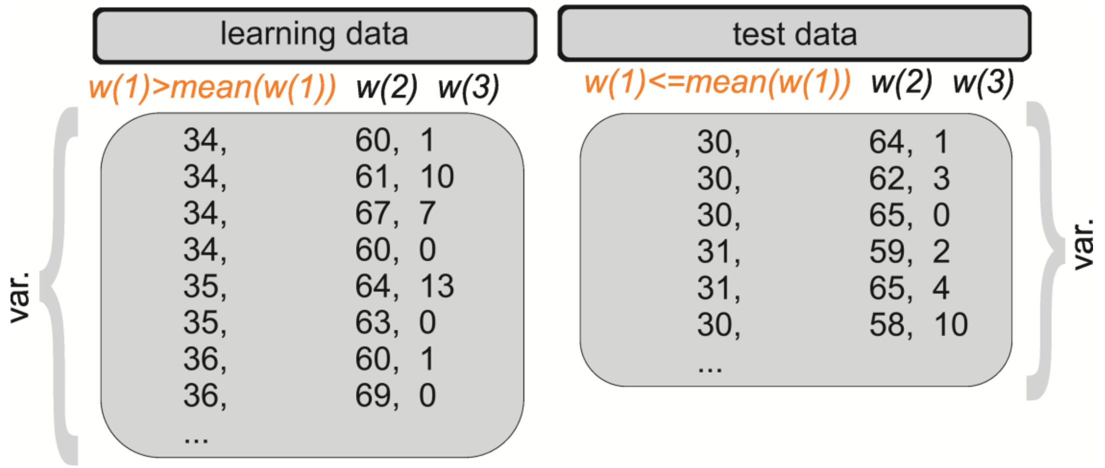

Fig. 12. Block diagram of division into the learning and test vector in terms of the value of *w*(1) feature.

Tab.2 Summary of accuracy values for different lengths of the learning and test vector divided by the mean of particular features *w*(1), *w*(2) or *w*(3).

**Type of classifier**	Size of the training data	Size of the test data	Feature division criterion	*ACC*
SVM	156	150	*w*(1)	71
Pruned decision tree	156	150	w(1)	65
Naive Bayes classifier	156	150	*w*(1)	73
SVM	140	166	*w*(2)	73
Pruned decision tree	140	166	*w*(2)	69
Naive Bayes classifier	140	166	*w*(2)	67

SVM	230	76	*w*(3)	49
Pruned decision tree	230	76	*w*(3)	51
Naive Bayes classifier	230	76	*w*(3)	51

Sixth example is linked to the influence of different length of the learning and test vector on the obtained results. The range of change in the length of the learning and test vector comprised the values from 20 to 300 cases and was changed every 2. The upper limit resulted from the maximum length of the data vector. The bottom limit instead resulted from the necessity to avoid the situation described in the first example. These vectors did not possess any common data. A total of 19 600 classifications were conducted for each type of the classifier. The obtained results for the three tested classifiers are shown in Fig. 13 - Fig. 18.

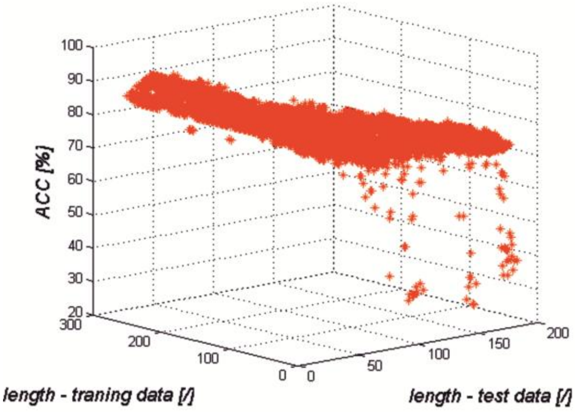

Fig.13. Graph of dependence of accuracy on the length of the learning and test vector for SVM

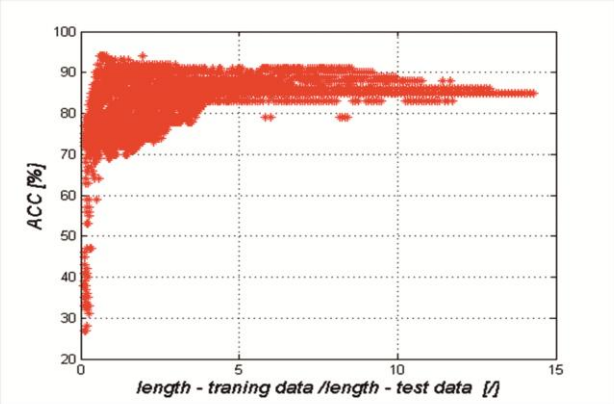

Fig. 14. Graph of dependence of accuracy (*ACC*) for various values of the ratio of the test and learning vector - SVM classifier

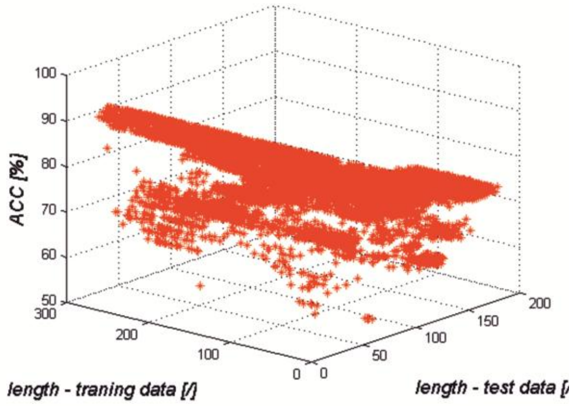

Fig.15. Graph of dependence of accuracy on the length of the learning and test vector for the pruned decision tree.

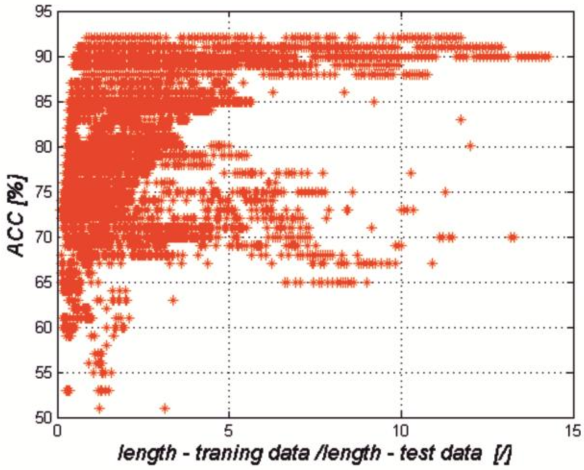

Fig. 16. Graph of dependence of accuracy (*ACC*) for various values of the ratio of the test and learning vector for the pruned decision tree

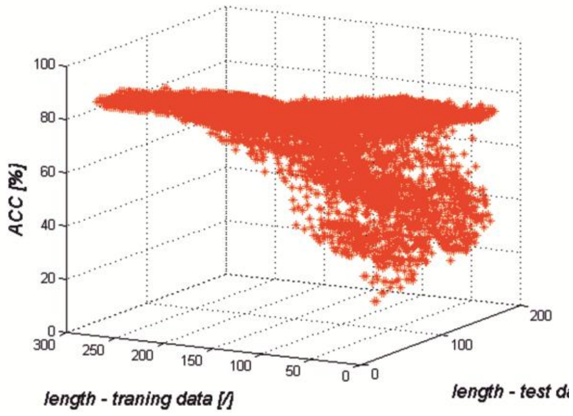

Fig.17. Graph of dependence of accuracy on the length of the learning and test vector for the naive Bayes classifier

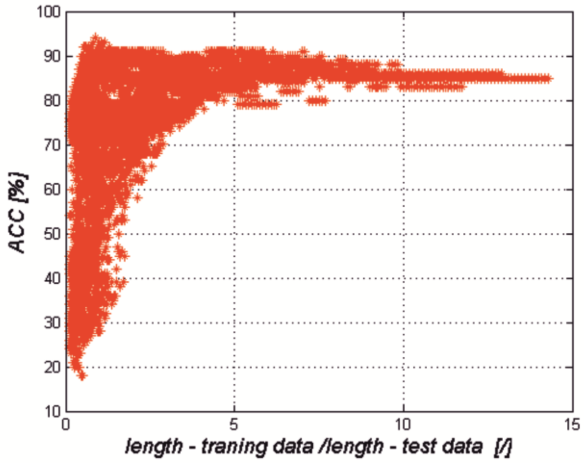

Fig. 18. Graph of dependence of accuracy (*ACC*) for various values of the ratio of the test and learning vector for the naive Bayes classifier

The above examples show limitations of machine learning. It is also quite possible to manipulate data in order to obtain better results. This quite important issue was not addressed in the reviewed book - even in a vague way (so as not to increase its volume excessively).

References

[1] Kenneth R Foster, Robert Koprowski and Joseph D Skufca: Machine learning, medical diagnosis, and biomedical engineering research - commentary BioMedical Engineering OnLine 2014, 13:94.

[2] Shachar Kaufman, Saharon Rosset, Claudia Perlich, Leakage in Data Mining: Formulation, Detection, and Avoidance, Proceedings of the 17th ACM SIGKDD international conference on Knowledge discovery and data mining Pages 556-563

[3] Haberman, S. J. (1976). Generalized Residuals for Log-Linear Models, Proceedings of the 9th International Biometrics Conference, Boston, pp. 104-122.

[4] Landwehr, J. M., Pregibon, D., and Shoemaker, A. C. (1984), Graphical Models for Assessing Logistic Regression Models (with discussion), Journal of the American Statistical Association 79: 61-83.
